# Horse Olfactory Exploration of Various Plants with Regard to Smell and Taste Familiarity

**DOI:** 10.3390/ani16060873

**Published:** 2026-03-11

**Authors:** Elżbieta Wnuk, Wiktoria Janicka, Anna Stachurska, Kamila Janicka, Marta Wnęk, Wojciech Jagusiak, Jarosław Łuszczyński

**Affiliations:** 1 Faculty of Animal Sciences and Bioeconomy, Department of Horse Breeding and Use, University of Life Sciences in Lublin, 13 Akademicka Street, 20-950 Lublin, Poland; elzbieta.wnuk@up.edu.pl (E.W.); anna.stachurska@up.edu.pl (A.S.); marta.wnek@up.edu.pl (M.W.); 2 Faculty of Animal Sciences and Bioeconomy, Institute of Biological Basis of Animal Production, University of Life Sciences in Lublin, 13 Akademicka Street, 20-950 Lublin, Poland; kamila.janicka@up.edu.pl; 3 Faculty of Animal Science, Department of Genetics, Animal Breeding and Ethology, University of Agriculture in Cracow, 21 Mickiewicz Avenue, 31-120 Cracow, Poland; wojciech.jagusiak@urk.edu.pl (W.J.); jaroslaw.luszczynski@urk.edu.pl (J.Ł.)

**Keywords:** equids, smell, known odour, known taste, unknown herbs, exploratory behaviour

## Abstract

The sense of smell is the first sense by which horses determine the suitability of feed for consumption. The aim of the study was to assess the influence of the degree of familiarity with the smell and taste of plants on horses’ olfactory exploration. Behavioural responses of twenty adult horses to nine herbs of three degrees of familiarity—least familiar (unknown in the first trial), known only by smell, and known by smell and taste—were investigated. During the tests, the horses were allowed to explore the individual herbs placed in a crib constructed to prevent them from eating the contents. Horses’ pre-consuming behaviour towards olfactory cues of plants was mainly expressed by different times of exploration. The horses explored herbs known by smell and taste less intensively than those initially unfamiliar, but not less intensively than herbs known only by smell. Thus, having more sensory experience (taste and olfactory) with a plant in the past, horses are often quicker to recognise plants based solely on the sense of smell in the future. The sex and type of the horse (warmblood, pony) may influence responses towards herbs of different levels of familiarity.

## Simple Summary

The senses of taste and smell are important in food selection and acceptance. Horses may exhibit a neophobic response to a novel odour or flavour, and they usually show preferences for known feed. The current study investigated if previous experience with the smell and taste of herbal plants influences horses’ olfactory exploration of those plants. The horses were allowed to smell nine herbs, three of which were of the least level of olfactory familiarity for them, three were known only by smell, and the remaining three were known by smell and taste. Herbs were placed in a special crib that prevented them from eating the plants. The horses paid more attention to the least familiar herbs, as indicated by the longest exploration times (sniffing, licking, nibbling, touching the crib), whereas the herbs known by smell and taste were explored for the shortest time. The taste experience plays a significant role in the olfactory recognition of plants. Prior olfactory exposure to a plant can induce a comparably rapid response to that seen in the case of taste–olfactory experience. Geldings and warmbloods are more cautious when exposed to the scent of less familiar plants and rely more on a previous taste experience.

## 1. Introduction

The sensory abilities of horses have evolved to support early danger recognition, which is essential for survival in a prey species [1]. Horses rely on various senses to perceive and respond to different stimuli in their environment [2], with a combination of visual, auditory, and likely olfactory cues as the most important in the detection of potential threats [3]. Odours may be considered one of the most salient environmental stimuli [4]. Therefore, they play a crucial role in eliciting context-specific behavioural responses [2,5]. Horses are thought to possess a highly developed sense of smell [6], which they employ in diverse behavioural contexts. These include social interactions [7], reproductive behaviour [8], predator avoidance [9] and foraging, particularly in evaluating food flavour [10].

Efficient foraging is critical for survival and reproductive success and is shaped both by the nature of available forage and the animal’s selective feeding capacity [11,12]. As grazing and browsing herbivores, horses evolved physiological and behavioural adaptations for consuming a fibrous, plant-based diet. They typically employ a patch foraging strategy, selecting from a wide range of plant species using various sensory inputs [13,14]. Diet selection is influenced by sensory characteristics such as visual cues, odour, taste, texture, availability, and variety [15,16]. Horses can selectively consume feeds to meet nutritional needs, favouring energy-rich options and adjusting the diet based on the nutrient and energy content of available plant species [16,17]. However, individual taste preferences may override nutritional value, leading horses to select foods based on palatability rather than health benefits [18]. Furthermore, previous experience with food, which may result in learned aversions or preferences and possibly post-ingestive nutrient feedback, is also crucial to dietary choices [10]. For example, it was observed that horses exhibit a strong preference for sweet (sucrose) solutions over sour, bitter, or salty alternatives, although the role of taste in regulating food intake remains incompletely understood [10].

Of the senses involved in foraging, olfaction appears to have the greatest influence, followed by taste [10,11]. Olfactory exploration in horses typically involves bringing the muzzle into close proximity to the odour source, allowing the animal to assess chemical cues [19]. Horses often explore feed through olfactory investigation before ingestion, and the presence of a familiar odour may enhance acceptance of novel food items [20]. Equines are sensitive to odours in their environment [21] and may discriminate between them. Rørvang et al. [19] demonstrated that horses were able to detect and distinguish between the odours of orange, peppermint, lavender, and cedarwood, with peppermint triggering the most pronounced exploratory and feeding-related responses. Understanding how horses respond to odours is crucial for predicting their reactions and ensuring safety for both the horses and handlers [2]. Olfactory stimulation, including novel scents and certain essential oils, has been proposed as an environmental enrichment strategy for domestic horses. In particular, lavender essential oil has been shown to reduce physiological and behavioural indicators of stress, while broader reviews suggest potential calming effects of olfactory enrichment [22].

Horses can form associations between specific scents and the corresponding tastes of food, even if they are initially unfamiliar with the taste itself [19,23]. Both the olfactory and gustatory senses are vital for food discrimination and preference. They function together with post-ingestive mechanisms to create learned associations between specific sensory cues and physiological outcomes, which can be either positive or negative [24]. However, despite the close relationship between taste and smell, little research has been conducted on the sensory sensitivity of these modalities in horses [1,2,9]. Based on the current literature, it remains unclear to what extent prior exposure to olfactory cues alone, as opposed to feeding experience, contributes to the olfactory recognition of a food during subsequent encounters. Although both olfaction and taste are known to play an important role in food selection in horses, olfaction is the primary sense providing herbivores with initial information about potential food sources. In the authors’ previous study [25], horses were shown to be capable of discriminating between previously unknown poisonous and non-poisonous plants based on the sense of smell alone. Therefore, olfactory exploration of food sources has clear biological significance. However, it has not yet been clarified whether familiarity with a plant’s odour alone is sufficient for early discrimination, or whether previous gustatory experience is necessary to enable recognition of the plant based solely on olfactory cues. Clarifying this question may contribute to a better understanding of how horses integrate sensory stimuli prior to ingestion when recognising and evaluating potential food sources.

The aim of the study was to assess the influence of familiarity with the smell and taste of plants on horses’ olfactory exploration and, further, to determine the importance of this familiarity in the discrimination of plants based on smell alone. We hypothesized that horses would explore plants they already knew by smell and taste less intensively, followed by plants known by smell alone, and most intensively those that were initially completely unfamiliar.

## 2. Materials and Methods

### 2.1. Horses

Twenty adult horses of mean age 9.75 ± 5.06 years, including 10 mares and 10 geldings, were involved in the study. Eleven of them were warmblood horses (Polish halfbred), and nine were ponies (Felin Pony). The horses had been kept at the same facility for at least three years. Veterinary checks with particular attention to horses’ general health, respiratory health, and digestive health was performed one week before and one week after the study. Additionally, horses were observed during daily handling by an experienced caretaker. All the horses included in the study were clinically healthy, without any signs of behavioural disorders, and had no respiratory system problems or allergies. The mares were not pregnant and not in oestrus.

All of the horses were maintained under similar conditions in individual box stalls (3 × 3 m) bedded with wheat straw. Box stalls were cleaned every day and the bedding was renewed every two weeks. A crib, hay feeder and an automatic drinker were installed in each box stall. The horses were fed in the morning, midday and evening with oats and meadow hay. Minerals were supplemented according to individual requirements. The horses were ridden usually for one hour daily. They were turned out into paddocks for approximately four hours before noon, where they had ad libitum access to hay.

### 2.2. Ethical Statement

The study was conducted at the facility where all the horses were kept for at least three years, which belonged to the University of Life Sciences in Lublin, Poland. The experiment did not cause them any pain, suffering or injury. The welfare of the horses was verified by a Panel of Experts on Animal Welfare (The Animal Welfare Team) in the Faculty of Animal Sciences and Bioeconomy of the university. The Animal Welfare Team stated that all procedures were conducted pursuant to the Polish Act of 15 January 2015 (amended on 17 November 2021) on the protection of animals used for scientific and educational purposes (Regulation of the Minister of Agriculture and Rural Development of 29 April 2022 on the minimum requirements to be met by the facility and the minimum requirements for the care of animals kept at the facility), and Directive 2010/63/EU of the European Parliament and of the Council of 22 September 2010 on the protection of animals used for scientific purposes. The study was approved by the above-mentioned Animal Welfare Team as non-invasive before the beginning (document No. ZdsDz/5/2024); therefore, further ethical approval was considered not applicable.

### 2.3. Plants

Based solely on the plant smell, the horses were to explore different dried herbs. At the beginning, nine herbs, not familiar to the horses, were chosen. Unfamiliarity was ensured by the fact that plants were not present in the feed or in any of the paddocks where the horses were turned out. The herbs used in the study were common horse supplements produced in Poland (Equiart Czesława Grycz, Kruszyna and Jar-Pasz, Jaworzno). They were randomly classified into one of the following groups (types) whose level of familiarity was altered during the study procedure:

- Least familiar (LF): fruit of black chokeberry (*Aronia melanocarpa*), seeds of caraway (*Carum carvi*), and leaves of raspberry (*Rubus idaeus*); plants with the lowest level of familiarity—completely unknown only during the first olfactory exposure;
- Known by smell (SM): fruit of dog rose (*Rosa canina*), flower of pot marigold (*Calendula officinalis*), and leaves of rosemary (*Salvia rosmarinus*); plants with a moderate degree of familiarity;
- Known by smell and taste (ST): yarrow herb and flower (*Achillea millefolium*), leaves of oregano (*Origanum vulgare*), and motherwort herb (*Leonurus cardiaca*); plants with the highest level of familiarity.

### 2.4. Procedure

**Figure 1:**
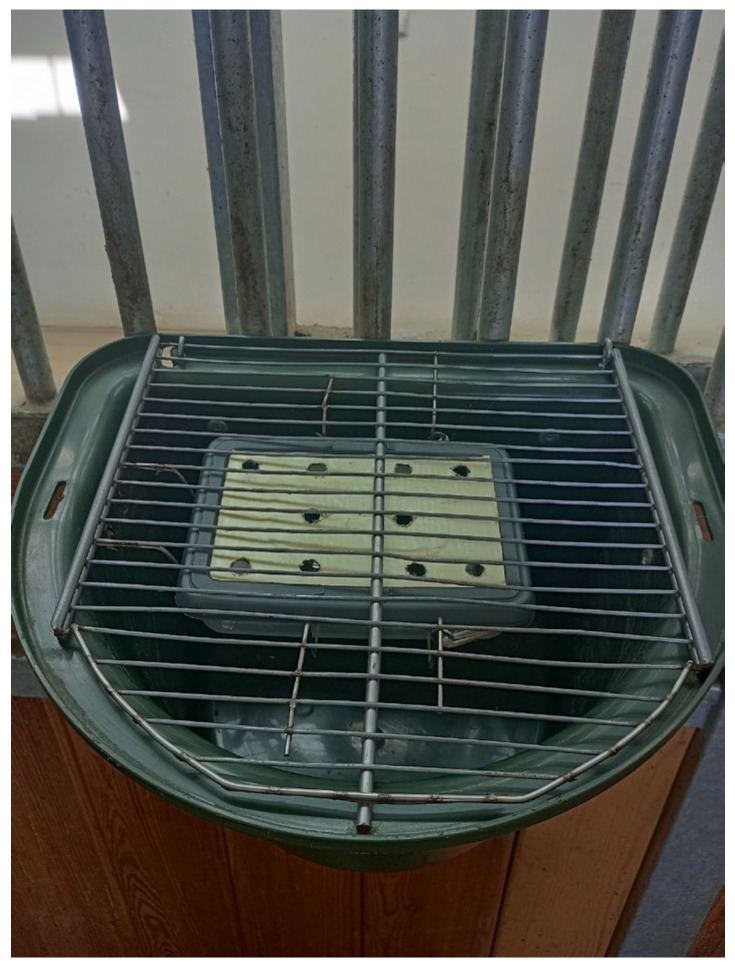
The experimental crib equipped with a plastic box containing plant material.

The study consisted of three stages: (1) habituation and positive conditioning of an experimental crib used in the study, (2) familiarising the horses with the smell and/or taste of selected herbs and (3) the main stage of the study—assessing olfactory exploration of the studied plants. The horses were tested in their own box stalls. During the tests, all horses remained in the stable, which was quiet; there were no noises, except for the two experimenters, no other people were present, and no other activities were carried out, such as cleaning or preparing feed. Otherwise, the horses’ attention could have been diverted, and they would not have shown interest in olfactory exploration. The experimental crib (39 cm long × 32.5 cm wide × 26 cm high) [25] was equipped with a plastic box (20 cm × 14 cm × 5 cm) with ten holes, each of 1.2 cm diameter in an opaque cover, allowing the smell of a plant placed inside to be perceived while preventing visual cues. A steel bar securing access to the crib was installed, with the plastic box attached directly underneath to prevent it from falling out or being manipulated by the horses (Figure 1). This crib was self-made at the university workshop and was not used for usual feeding and was only used for the purposes of the study. It was hung in the middle of the frontal wall of the box stall on its inner side at the height of 120 cm for warmblood horses and 100 cm for ponies, approx. 1.5 m from the usual crib (hung in the corner of the box stall) and approx. 1.5 m from the neighbouring box stalls. This aimed to minimise the risk of neighbouring horses being additionally exposed to the scent of the herbs. Although the procedure of testing was recorded in situ by the experimenters to enable a review of the data, it was also recorded by a camera (Panasonic HC-V180, Amsterdam, The Netherlands). The camera was placed on a mobile tripod in the stable passage at a height of 145 cm (120 cm for ponies), 1 m from the front of the successive box stall doors opposite the crib.

#### 2.4.1. Stage 1

Prior to the study, the horses were conditioned to approach and touch the crib. It was a requirement enabling the experiment to be conducted—horses had to be motivated to perform these actions; otherwise, they would not detect the scent of the plants placed in the crib during later stages of the study. During conditioning, the box placed in the crib was empty, without a plant. Two experimenters participated in each stage of the study, and their roles were as follows: Experimenter 1 (KJ) entered the box stall, held a horse in hand, led it to the opposite wall and positioned the horse facing the front wall of the box stall using a lead rope attached to the halter. Simultaneously, Experimenter 2 (MW) hung the crib in the middle of the frontal box stall wall, then left the box stall and turned on a camera. Experimenter 1 ensured that the horse’s head was facing the crib at a distance of 1 m (starting position) and then released the horse and left the box stall.

The task in this stage involved a horse independently approaching and touching it, for which it was rewarded with a handful of oats. If the horse failed to do so within 10 s, the experimenter led it to the crib and encouraged it to touch the crib by scratching the steel bar. Most of the horses were introduced to the above task during earlier studies [25,26] and willingly approached and touched the crib in the first trial. However, to ensure a high level of motivation to approach the crib, all horses underwent this task three times. If, during the third trial (a control task), a horse independently approached the crib within 10 s and touched it, it was considered to have successfully completed the conditioning process and was allowed to participate in subsequent stages (Stage 2, Stage 3). All of the horses passed a control task and were included in further stages. In addition, in order to maintain the horses’ interest in the crib during the main stage (Stage 3), a reminder trial of approaching was conducted throughout the study once a day, always after all horses had been tested.

#### 2.4.2. Stage 2

Next, the horses were exposed to the smell of the SM herbs, which were placed successively into the box in the crib. Each horse was encouraged to explore and smell the box containing 30 g of herb by being led towards the crib by one of the experimenters. Olfactory exploration of a given plant was indicated by behaviours such as touching a steel bar of the crib with the horse’s nostrils, licking and/or nibbling it, or clearly flaring its nostrils positioned directly above the bar, as observed in previous studies [25,26]. If the horse did not lower its head to the crib and begin exploring it, the experimenter encouraged the horse to do so by scratching the steel bar and gently tugging the lead rope. Horses were allowed to explore the crib for a maximum of 30 s to avoid habituation to the smell and the crib, as well as a loss of motivation to approach it later. However, at least 5 s of continuous exploration was required. The horses were subjected to the scent of each of the three herbs once a day, one by one, every 20 min, for six successive days. It was assumed that the horses had become familiar with the scent of each plant if, during at least the last three exposures, they independently lowered their heads over the crib and explored it continuously for at least 5 s. All horses met this criterion. Importantly, the horses did not exhibit the mentioned behaviours during Stage 1 (crib without plants) and only approached and touched the crib. Thus, the horses were attracted by the scent of the herbs.

Similarly, the horses became familiar with the smell and taste of three ST herbs: they were fed 100 g of oats with an addition of 10 g of each herb, one by one, every 30 min, from their usual mangers, during the same six days. The horses voluntarily approached the crib once the oats with herb were put into the manger. The horses were subjected to the procedure of familiarisation with the odour and taste of the herbs always after the consumption of concentrate feed (at least 30 min post-feeding): they consumed the ST herbs at 7:00–8:30 a.m., while they were accustomed to the smell of SM herbs at 13:00–14:30 p.m. The LF plants were not presented. Each herb was presented in a separate box. The plant material was placed in the box immediately before exposure to the horse and replaced for each horse. After the horse had finished exploring the crib, the steel bar of the crib and the lid of the box were washed (using neutral unscented detergent), rinsed thoroughly with warm running water and then wiped dry with a disposable paper towel, to limit the transfer of scent from one horse to another [4]. Each time these items were dried and the box of herbs was placed for the next horse, new disposable gloves were worn.

#### 2.4.3. Stage 3

**Figure 2:**
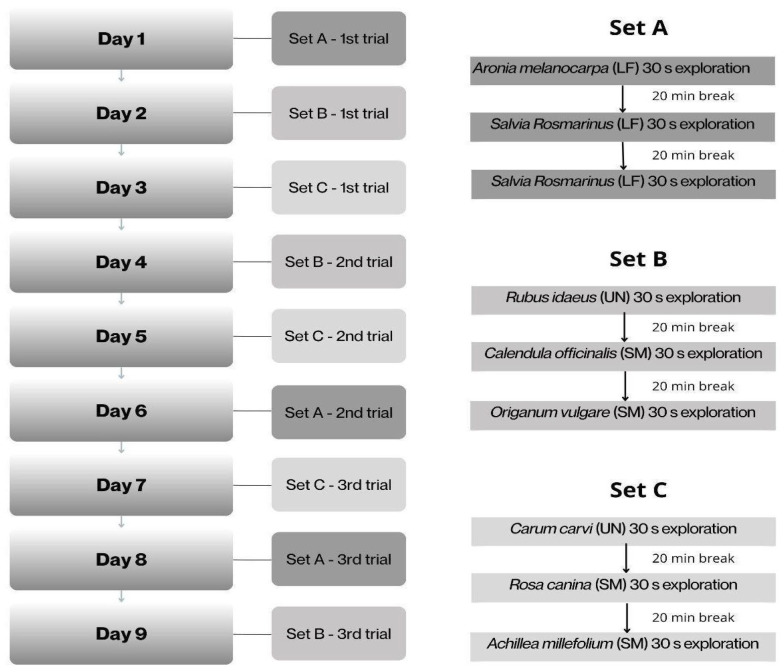
Course of Stage 3 of the experiment.

For the purpose of the study, the plants were randomly classified into three sets, each containing one LF, one SM and one ST herb: A—*Aronia melanocarpa*, *Salvia rosmarinus*, *Leonurus cardiaca*; B—*Rubus idaeus*, *Calendula officinalis*, *Origanum vulgare*; C—*Carum carvi*, *Rosa canina*, *Achillea millefolium*. Since one set was presented to the horses three times, one set in one day, the test lasted nine days for a horse. The horses were subjected to the scent of each of the three herbs, one by one, every 20 min and were allowed to explore each of them for 30 s (Figure 2). The experiment was conducted between 13:00 and 15:00 p.m., beginning at least 30 min after the horses’ midday feeding in order to avoid disrupting their feeding routine and to minimise the potential influence of food anticipation on olfactory exploration. Although the LFs (the lowest level of familiarity) became somewhat familiar in the second and third trials, SM (moderate level of familiarity) and ST (the highest level of familiarity) were considerably different since horses were familiarised with them six times prior to the study. The order of the sets and herbs in a set was changed. For each test, 30 g of a dried herb was placed in a clean, separate plastic box away from the stable to ensure the odour would not be accessible to the horses. The plastic box was then placed in the experimental crib, which was hung in the box stall during testing. The plant material was replaced for each horse (each herb had a separate container) and the steel bar of the crib and the lid of the box were cleaned according to the procedure of Stage 2.

### 2.5. Variables

The 30 s observation began when the horse was released from the starting position (described in Stage 1), moved forward, approached the crib, and the timing started at first contact when the muzzle was approx. 5 cm away from the crib. The latency to approach the crib was excluded from the 30 s observation period. Based on the authors’ previous studies [25,26], the following variables were recorded:

- Exploration time (s)—the total time of interaction with a crib, e.g., sniffing, touching, licking, nibbling, measured with an accuracy of 1 s.
- Exploration frequency—the total number of consecutive exploration events. Those separated by a break of a minimum of 3 s were counted separately.
- Prevalence of the following specific behaviours during the crib exploration: nibbling a crib—moving the upper lip upward and downward against an object with the jaws closed, associated with an investigative approach to the object [27]; licking a crib—contact between the horse’s tongue and the crib [28]; mouth licking—visible tongue licking the lips without making contact with the crib; empty-mouth chewing—moving the lower jaw in a chewing motion without any food in the mouth [29].

### 2.6. Statistical Analysis

Analyses were performed using the GLIMMIX procedure in SAS, 9.4 version (SAS Institute Inc., Cary, NC, USA). Data were analyzed using generalized linear mixed models (GLMMs) fitted with either a Poisson or negative binomial distribution, depending on data dispersion. A single random effect for horse that accounted for within-cluster correlation was applied, while fixed effects included sex, trial, horse type, plant species and plant type. In the authors’ previous study, it was found that horses of different types (ponies, warmbloods) and sexes may exhibit certain differences in olfactory sensitivity to a positively conditioned odour [26]. Therefore, it was decided to include the influence of these factors in the current study. Overdispersion was evaluated using the Pearson chi-square statistic divided by its degrees of freedom. For most traits, equidispersion justified a Poisson specification, whereas the only overdispersed trait—“time of exploration”—was modeled using a negative binomial distribution. Significant effects of the studied factors were further explored by comparing least squares means across levels with Tukey–Kramer adjustment. The results were presented in tables as raw means, standard deviations and minima and maximum==a. The differences between the means were considered significant at *p* < 0.05 (marked with the same small letters in tables) and highly significant at *p* < 0.01 (marked with the same capital letters in tables); letters refer to GLMM LSMeans comparisons. The “n” value in tables refers to the number of observations (repeated measures), not the number of horses. Within-horse correlation is handled by the random horse effect in the GLMM.

## 3. Results

### 3.1. Effect of Plant Type on Horses’ Exploration

The horses explored the experimental crib containing a sample of a plant known to them by smell and taste (ST) for the shortest time; however, a significant difference was found only between LF and ST (Table 1). No significant differences were found in the frequency of exploration and in the frequency of specific behaviours: crib nibbling, crib licking, mouth licking and empty-mouth chewing with regard to the plant type (Table 1).

When analysing the effect of gender on the horses’ response to LF, SM and ST, no significant differences were found in the exploration time and frequency between geldings and mares (Table 2). Considering the variables across the different plant types, the geldings explored ST plants for a shorter time than LF plants (*p* < 0.05). In mares, similar tendencies were observed, although the differences were not statistically significant.

Two significant differences between mares and geldings in the frequency of specific behaviours were found (Table 3). Geldings licked the crib and mouth more often than mares when SM herbs were exposed (*p* < 0.05).

No significant differences were found between warmblood horses and ponies in the time and frequency of exploration of particular plant types (Table 4). A trend of shorter exploration time for ST compared to LF was statistically significant in warmblood horses (*p* < 0.05), whereas it was not significant in ponies.

There were no significant differences between horse types in the occurrence of specific behaviours and within each horse-type group for LF, SM and ST (Table 5).

Taking into account the effect of subsequent trials in the group of plants with the lowest level of familiarity (LF), a significant difference was found only for the exploration time variable (Table 6). It was observed that horses explored them significantly longer in the first trial compared to the second and third trials (*p* < 0.01), but no differences in exploration time were found between the second and third trials.

### 3.2. Effect of Plant Species on Horses’ Exploration

It was found that *Aronia melanocarpa* was explored significantly longer than that of *Rubus idaeus*, *Leonurus cardiaca*, *Origanum vulgare*, *Salvia rosmarinus* (*p* < 0.01) and *Achillea millefolium* (*p* < 0.05; Table 7). The mean frequency of exploration did not significantly differ between the plant species.

Of the herbs studied, exposure of horses to *Aronia melanocarpa* resulted in more frequent crib licking than exposure to *Rubus idaeus*, *Calendula officinalis*, *Origanum vulgare and Leonurus cardiaca* (Table 8). No significant differences were found for other behaviours.

## 4. Discussion

Although food choice is determined by nutrients, different food sensory characteristics (smell, taste and texture) and post-ingestive feedback (positive or negative) [10], olfaction allows the first information to be perceived about the potential food. Consuming plants through sampling is an important mechanism in the association between the sensory properties of food and its metabolic consequences, resulting, among others, from its nutritional value [13]. As shown in our previous study [25], horses are able to gather information about the edibility of plants based on olfactory cues alone.

In the current study, the horses’ olfactory exploration of plants of different levels of sensory familiarity was compared. More specifically, animals were exposed to the smell of three groups of herbs: initially unfamiliar (the lowest level of familiarity; completely unfamiliar in the first trial—LF) and known by smell (moderate level of familiarity; after familiarising the horses with the smell—SM), as well as known by smell and taste (the highest level of familiarity—ST). As predicted, the horses explored the LF for the longest time, followed by SM and ST. However, a significant difference in the time of exploration was found only between LF and ST. Hence, it can be suggested that the horses’ olfactory exploration varies to some degree depending on the level of familiarity with a plant, which is discussed in detail as follows.

It was demonstrated that the sense of smell plays a key role in horses’ feed intake [30] and is important in the first interactions with a novel food. A previous study [20] showed that horses did not smell (or smelled briefly) the food they knew very well by smell and taste, i.e., oats. This finding corresponds to the fact that the horses in the current study also explored ST for the shortest time, followed by SM. More sensory experience shortened the time of exploration of the plant in subsequent encounters, probably due to its faster recognition. Simultaneously, less experience, as in the case of LF herbs, may need more cautious exploration. Hence, more time spent exploring LF plants compared to ST may have been due to the need to gather information on a plant that the horses had not yet encountered or had encountered only rarely. Given the risk of coming across unpalatable, pernicious or poisonous plants on pasture [31], it is crucial for horses to collect as much information as possible based on the sense of smell. Previous studies have shown that olfactory exploration decreases in successive encounters [4,25]. Hence, it is possible that with greater experience of an olfactory cue, animals may require less time to recognize an odour in the future.

However, it should be noted that the main analysis of the current study focused on comparing the responses to the LF, SM, and ST plants based on the three trials. As shown in the authors’ previous study [25], the exploration time of both poisonous and non-poisonous plants decreased gradually and significantly in subsequent trials. The reason for this may not have been solely because of faster recognition with growing olfactory experience, but also due to gradual habituation to the odours, as reported by Rørvang et al. [4], or a lower motivation to participate in the tests, as shown by Mal et al. [32]. In the current study, the number of trials was particularly important for LF plants, which were classified as those of the lowest level of familiarity. Horses explored LF plants for the longest in the first trial, when they were completely unknown to the horses, and for the shortest in the third trial, when they became somehow familiar. Therefore, it is possible that the horses learned to recognise the scent of a new plant on the first attempt and did not need to explore the plant as thoroughly on the subsequent attempt. This may also be suggested by the fact that, when considering all three trials, no significant differences were found in the olfactory exploration of SM and LF plants. This observation may simultaneously somehow limit the assumption that greater familiarity with the smell of a plant alone reduces the duration of olfactory exploration upon subsequent exposure. Unlike in the previous study [25], only edible herbs for horses—not poisonous—were used. This may explain why the horses explored LF and SM for a similar amount of time: the scent of both herb types did not alert the horses to the presence of poison. Hence, it may be suggested that knowing a non-poisonous plant solely by smell does not affect how quickly horses recognise it. However, the reasons for this observation may also be completely different, as in the previously mentioned studies [4,26,32]. It therefore seems reasonable that in future studies, when assessing horses’ reactions to plants with varying degrees of familiarity, only the first trial, or consecutive trials, treated independently, should be included in the comparisons. The comparison of the horses’ exploratory behaviour from all three trials may be a potential limitation of our study and should be taken into account when analysing the results of this work. Further, as observed, the first olfactory impression is of importance for horses, since it is explored the most thoroughly. This may have practical implications, as after just one exposure to a new scent, a horse can develop a positive or negative attitude toward it.

The above-mentioned observation, combined with the significantly shorter exploration time of ST compared to LF, may support the importance of the sense of taste in the olfactory recognition of encountered plants. It can be suggested that the more sensory experience (smell and taste) horses had with a plant in the past, the faster they recognised the plant based solely on their sense of smell in the future. Van den Berg et al. [24] previously noted that pre-ingestive cues, like the smell and taste of foods, are crucial in diet selection and that volatiles and olfaction may be important factors when horses choose between familiar and unfamiliar foods [13]. Another reason why the horses took the shortest time to sniff the herbs they had previously eaten during the learning phase (ST) may be the post-consumption effect. Since the herbs used in the present study were commonly intended for horses as supplements (were edible), the studied individuals probably experienced positive, or at least neutral, post-ingestive feedback in the case of becoming familiar with ST. According to other authors [31,33,34], previous experience is crucial for conditioned food preferences or aversions and, hence, plays an important role in response to a re-encounter with the plants. However, no differences were observed in the duration of olfactory exploration between plants known only by scent, with which the horses had been previously familiarized (SM), and those known by both taste and scent (ST). This fact could, on the one hand, limit the role of the sense of taste in olfactory exploration and in the recognition of non-toxic plants through smell. On the other hand, as previously mentioned, horses may have quickly habituated to odours to which they were repeatedly exposed or have lost interest in them [4,32], and hence, no differences between SM and ST were observed. These results may have practical implications for introducing new foods into the diet of horses, where prior exposure to the odour could reduce neophobic responses [24]. However, further research would be required to confirm the effects on feeding behaviour following preliminary olfactory explorations.

Practically, the only variable for which significant differences were observed among the factors studied (plant type, plant species, biological traits) was the duration of olfactory exploration. No significant differences, with a few exceptions for sex and plant species factors, were found in the frequency of exploration or in the occurrence of specific behaviours, i.e., crib nibbling, crib licking, mouth licking and empty-mouth chewing between the exploration of various types of herbs. Therefore, it may be suggested that these behaviours have lesser importance as indicators of the pre-consuming explorative behaviour of horses, which is in accordance with the authors’ previous studies [25,26].

Horses’ responses towards certain species of herbs varied only in a few cases between *Aronia melanocarpa* and five other herbs in the time of exploration, and between *Aronia melanocarpa* and four other herbs in the frequency of crib licking. In those cases, horses explored *Aronia melanocarpa* for the longest and showed crib licking the most often. This herb was classified as LF, so on the one hand, this may result from a more thorough exploration of a plant with a low level of familiarity. However, on the other hand, the results may indicate that this herb was more attractive to horses and that explorative behaviour towards different plant species was influenced by individual preferences. This is in accordance with the observations made by Rørvang et al. [19], who showed that despite the horses’ ability to distinguish between four different odours, they expressed significantly more interest when exposed to one of them. Moreover, in our previous study [25], differences were observed in the exploration time of different non-poisonous plant species, which could indicate that they were not equally attractive to horses. Horses are not unique in this regard; other herbivorous species also have their own preferences regarding the flavour or type of food. For example, dairy cows may prefer vanilla or fenugreek flavours when given a choice of a new variety of feed [35]. Sheep proved to be more selective than cattle when choosing the most frequently consumed plant from among several species available in the pasture sward [36]. Therefore, the impact of the studied species of plants and possibly individual preferences of the horses on the results of the current study cannot be excluded. This is a potential limitation, although it would not be possible to select plants of equal attractiveness to horses. It would require prior exposure of the test horses to different herb species, which, in turn, would affect future results. A possible solution could be to use more plant species in future studies.

Regarding the effect of the studied biological traits of the horses (sex, horse type) on responses to herbs of different degrees of familiarity, no differences were observed between warmbloods and ponies, and only in two cases were sex-related differences observed: geldings licked the crib and their mouths significantly more often than mares when exploring SM. The overall differentiation in exploring various plant types resulted only from differences found within geldings and warmblood horses. In these cases, the tendencies were similar to those observed for all the studied horses: geldings and warmbloods explored LF significantly longer than ST. The outcomes suggest that for geldings and warmbloods, the degree of familiarity with a plant may be more important than for mares and ponies, for which no differences were observed. These horses are more cautious when exposed to the scent of less familiar plants (LF) and rely more on a previous taste experience. Similarly, in the study conducted by Wnuk et al. [26], differences related to sex and horse type in the exploration of the familiar mint odour were infrequent, and the majority of significant differences observed in response to varying odour concentrations reflected within-group variations. Rørvang et al. [30] previously noted that the horses’ olfactory interest in different plant odour oils varied with age and gestational status, but not sex. The rare differences in responses between geldings and mares in the current study can be cautiously interpreted as a result of their role in a herd. Since a dominant mare decides on the direction of the march and the time of rest under natural conditions [37], it cannot be excluded that mares can also decide to consume plants based on the sense of smell more quickly. More acute olfaction in females than males could also be due to the role olfactory cues play in mother–offspring recognition [38]. However, the few significant differences between horses of different sexes in the present study do not justify stating that there is a sex difference in olfactory ability. Considering the more varied olfactory exploration of warmblood horses compared to ponies according to the degree of familiarity, it is possible that the differences may be type- and/or breed-specific. Janczarek et al. [39] found that purebred Arabian horses showed the greatest variety in pellet taste preferences, whereas primitive horses were more cautious (hesitating, sniffing) before consumption. In summary, in light of the obtained results, it is suggested that variables such as sex and breed type should be taken into account when investigating horses’ responses to food-related odours and tastes. This will facilitate interpretation and enhance the transparency of findings across different studies. Considering differential responses depending on these biological factors may be useful not only in equine nutritional research, but also in the development of horse care products or aromas used for environmental enrichment.

## 5. Conclusions

Horses’ pre-consuming behaviour towards olfactory cues of plants is mainly expressed by different times of exploration. The horses explore herbs known to them by smell and taste less intensively than herbs that were initially unfamiliar. The taste experience may be of importance in the olfactory recognition of encountered plants. However, having more olfactory experience with a plant in the past may result in a similarly quick response as observed in taste–smell experience. Exploring the least familiar food for a longer time may be due to the need to gather information on a plant that the horses had not yet encountered or rarely encountered. Horses explore unfamiliar plants for the longest duration in the first trial, which may reflect greater caution upon first encounter, rapid recognition of the odour in subsequent trials, or quick habituation leading to decreased interest in the odour. The sex and type of the horse (warmblood, pony) may, to some degree, influence responses towards herbs of different degrees of familiarity. Geldings and warmbloods are more cautious, particularly when less familiar with a plant, and then explore it for longer. Further studies should include more species of plants to reduce the possible impact of varying species’ attractiveness on the results. It is further suggested to compare horses’ responses to plant odours of varying familiarity exclusively in the first trial.

## Figures and Tables

**Table 1:** Overall time [s], frequency [number per 30 s] of exploration and frequency of specific behaviours (crib nibbling, crib licking, mouth licking, empty-mouth chewing) [number per 30 s] with regard to different plant types.

Plant Type	n	Mean	SD	Min	Max
Time of exploration					
LF	180	11.6 ^a^	7.41	1.0	30.0
SM	180	10.8	6.79	1.0	30.0
ST	180	9.7 ^a^	6.86	1.0	30.0
Frequency of exploration					
LF	180	1.9	0.77	1.0	4.0
SM	180	2.0	0.84	1.0	4.0
ST	180	1.8	0.72	1.0	4.0
Crib nibbling					
LF	180	0.5	0.74	0.0	3.0
SM	180	0.4	0.68	0.0	3.0
ST	180	0.4	0.67	0.0	4.0
Crib licking					
LF	180	0.3	0.52	0.0	2.0
SM	180	0.2	0.49	0.0	2.0
ST	180	0.2	0.45	0.0	3.0
Mouth licking					
LF	180	0.1	0.30	0.0	2.0
SM	180	0.1	0.30	0.0	2.0
ST	180	0.1	0.30	0.0	2.0
Empty-mouth chewing					
LF	180	0.3	0.52	0.0	2.0
SM	180	0.2	0.45	0.0	2.0
ST	180	0.2	0.51	0.0	2.0

n—number of observations (repeated measures); SD—standard deviation; LF—least familiar plants; SM—plants known by smell; ST—plants known by smell and taste. Differences between means in columns are statistically significant: they are marked with the same small letters (^a^) at *p* < 0.05. Letters refer to GLMM LSMeans comparisons.

**Table 2:** Mean time of exploration [s] and frequency of exploration [number per 30 s] with regard to plant type and horse gender.

Plant Type	Mares					Geldings				
	n	Mean	SD	Min	Max	n	Mean	SD	Min	Max
Time of exploration										
LF	90	11.2	7.39	1.0	30.0	90	12.0 ^a^	7.46	1.0	29.0
SM	90	10.1	6.99	1.0	30.0	90	11.5	6.54	2.0	30.0
ST	90	9.6	6.65	1.0	30.0	90	9.9 ^a^	7.10	2.0	30.0
Frequency of exploration										
LF	90	1.8	0.81	1.0	4.0	90	1.9	0.74	1.0	4.0
SM	90	1.9	0.85	1.0	4.0	90	2.1	0.82	1.0	4.0
ST	90	1.8	0.68	1.0	3.0	90	1.8	0.76	1.0	4.0

n—number of observations (repeated measures); SD—standard deviation; LF—least familiar plants; SM—plants known by smell; ST—plants known by smell and taste. Differences between means in columns are statistically significant: they are marked with the same small letters (^a^) at *p* < 0.05. Letters refer to GLMM LSMeans comparisons.

**Table 3:** Mean frequency of specific behaviours (crib nibbling, crib licking, mouth licking, empty-mouth chewing) [number per 30 s] with regard to plant type and horse gender.

Plant Type	Mares					Geldings				
	n	Mean	SD	Min	Max	n	Mean	SD	Min	Max
Crib nibbling										
LF	90	0.5	0.71	0.0	3.0	90	0.5	0.77	0.0	3.0
SM	90	0.4	0.65	0.0	2.0	90	0.5	0.71	0.0	3.0
ST	90	0.4	0.72	0.0	4.0	90	0.4	0.61	0.0	2.0
Crib licking										
LF	90	0.2	0.48	0.0	2.0	90	0.3	0.55	0.0	2.0
SM	90	0.1 ^x^	0.41	0.0	2.0	90	0.3 ^x^	0.55	0.0	2.0
ST	90	0.2	0.39	0.0	1.0	90	0.2	0.50	0.0	3.0
Mouth licking										
LF	90	0.1	0.32	0.0	2.0	90	0.1	0.29	0.0	1.0
SM	90	0.0 ^x^	0.18	0.0	1.0	90	0.1 ^x^	0.37	0.0	2.0
ST	90	0.1	0.27	0.0	1.0	90	0.1	0.32	0.0	2.0
Empty-mouth chewing										
LF	90	0.3	0.51	0.0	2.0	90	0.2	0.53	0.0	2.0
SM	90	0.2	0.47	0.0	2.0	90	0.2	0.43	0.0	2.0
ST	90	0.4	0.59	0.0	2.0	90	0.2	0.40	0.0	2.0

n—number of observations (repeated measures); SD—standard deviation; LF—least familiar plants; SM—plants known by smell; ST—plants known by smell and taste. Differences between means in rows are statistically significant: they are marked with the same small letters (^x^) at *p* < 0.05. Letters refer to GLMM LSMeans comparisons.

**Table 4:** Mean time of exploration [s] and frequency of exploration [number per 30 s] with regard to plant and horse types.

Plant Type	Warmblood Horses					Ponies				
	n	Mean	SD	Min	Max	n	Mean	SD	Min	Max
Time of exploration										
LF	99	12.1 ^a^	7.76	1.0	30.0	81	10.9	6.96	1.0	29.0
SM	99	11.2	6.89	2.0	30.0	81	10.4	6.68	1.0	30.0
ST	99	10.1 ^a^	7.54	2.0	30.0	81	9.3	5.94	1.0	22.0
Frequency of exploration										
LF	99	1.9	0.75	1.0	4.0	81	1.9	0.81	1.0	3.0
SM	99	2.0	0.85	1.0	4.0	81	2.0	0.83	1.0	4.0
ST	99	1.7	0.74	1.0	4.0	81	1.8	0.69	1.0	3.0

n—number of observations (repeated measures); SD—standard deviation; LF—least familiar plants; SM—plants known by smell; ST—plants known by smell and taste. Differences between means in columns are statistically significant: they are marked with the same small letters (^a^) at *p* < 0.05. Letters refer to GLMM LSMeans comparisons.

**Table 5:** Mean frequency of specific behaviours (crib nibbling, crib licking, mouth licking, empty-mouth chewing) [number per 30 s] with regard to plant and horse types.

Plant Type	Warmblood Horses					Ponies				
	n	Mean	SD	Min	Max	n	Mean	SD	Min	Max
Crib nibbling										
LF	99	0.5	0.77	0.0	3.0	81	0.5	0.69	0.0	2.0
SM	99	0.4	0.67	0.0	2.0	81	0.5	0.69	0.0	3.0
ST	99	0.3	0.54	0.0	2.0	81	0.5	0.78	0.0	4.0
Crib licking										
LF	99	0.3	0.57	0.0	2.0	81	0.2	0.44	0.0	2.0
SM	99	0.2	0.48	0.0	2.0	81	0.3	0.51	0.0	2.0
ST	99	0.2	0.47	0.0	3.0	81	0.2	0.42	0.0	1.0
Mouth licking										
LF	99	0.1	0.33	0.0	2.0	81	0.1	0.26	0.0	1.0
SM	99	0.1	0.33	0.0	2.0	81	0.1	0.24	0.0	1.0
ST	99	0.1	0.24	0.0	1.0	81	0.1	0.35	0.0	2.0
Empty-mouth chewing										
LF	99	0.3	0.54	0.0	2.0	81	0.3	0.50	0.0	2.0
SM	99	0.2	0.43	0.0	2.0	81	0.2	0.47	0.0	2.0
ST	99	0.2	0.50	0.0	2.0	81	0.3	0.52	0.0	2.0

n—number of observations (repeated measures); SD—standard deviation; LF—least familiar plants; SM—plants known by smell; ST—plants known by smell and taste. No significant differences were found.

**Table 6:** Mean time of exploration of herbs in the least familiar group in subsequent trials.

Trial	n	Mean	SD	Min	Max
Time of exploration					
1	60	14.85 ^AB^	7.19	3.00	28.00
2	60	9.90 ^A^	8.25	1.00	30.00
3	60	10.05 ^B^	5.56	1.00	27.00

n—number of observations (repeated measures); SD—standard deviation. Differences between means in columns are statistically significant: they are marked with the same capital letters (^A^, ^B^) at *p* < 0.01. Letters refer to GLMM LSMeans comparisons.

**Table 7:** Mean time [s] and frequency [number per 30 s] of exploration for different plant species.

Plant Species	n	Mean	SD	Min	Max
Time of exploration					
*Aronia melanocarpa* (LF)	60	15.0 ^ABCDa^	8.15	1.0	30.0
*Carum carvi* (LF)	60	10.7	6.55	1.0	29.0
*Rubus idaeus* (LF)	60	9.1 ^A^	6.19	2.0	27.0
*Rosa canina* (SM)	60	11.5	7.67	2.0	30.0
*Calendula officinalis* (SM)	60	11.3	6.10	3.0	30.0
*Salvia rosmarinus* (SM)	60	9.6 ^D^	6.44	1.0	27.0
*Achillea millefolium* (ST)	60	10.6 ^a^	6.83	2.0	26.0
*Origanum vulgare* (ST)	60	9.5 ^C^	6.22	2.0	30.0
*Leonurus cardiaca* (ST)	60	9.1 ^B^	7.49	1.0	30.0
Frequency of exploration					
*Aronia melanocarpa* (LF)	60	2.1	0.84	1.0	4.0
*Carum carvi* (LF)	60	1.8	0.74	1.0	3.0
*Rubus idaeus* (LF)	60	1.8	0.72	1.0	3.0
*Rosa canina* (SM)	60	1.8	0.78	1.0	4.0
*Calendula officinalis* (SM)	60	2.1	0.84	1.0	4.0
*Salvia rosmarinus* (SM)	60	2.1	0.88	1.0	4.0
*Achillea millefolium* (ST)	60	1.9	0.74	1.0	3.0
*Origanum vulgare* (ST)	60	1.8	0.70	1.0	4.0
*Leonurus cardiaca* (ST)	60	1.7	0.71	1.0	3.0

n—number of observations (repeated measures); SD—standard deviation; LF—least familiar plants; SM—plants known by smell; ST—plants known by smell and taste. Differences between means in columns are statistically significant: they are marked with the same small letters (^a^) at *p* < 0.05 and with the same capital letters (^A^, ^B^, ^C^, ^D^) at *p* < 0.01. Letters refer to GLMM LSMeans comparisons.

**Table 8:** Mean frequency of specific behaviours (crib nibbling, crib licking, mouth licking, empty-mouth chewing) [number per 30 s] with regard to different plant species.

Plant Species	n	Mean	SD	Min	Max
Crib nibbling					
*Aronia melanocarpa* (LF)	60	0.8	0.79	0.0	3.0
*Carum carvi* (LF)	60	0.4	0.66	0.0	3.0
*Rubus idaeus* (LF)	60	0.4	0.69	0.0	2.0
*Rosa canina* (SM)	60	0.4	0.62	0.0	2.0
*Calendula officinalis* (SM)	60	0.4	0.74	0.0	3.0
*Salvia rosmarinus* (SM)	60	0.6	0.67	0.0	2.0
*Achillea millefolium* (ST)	60	0.4	0.72	0.0	4.0
*Origanum vulgare* (ST)	60	0.5	0.72	0.0	2.0
*Leonurus cardiaca* (ST)	60	0.4	0.55	0.0	2.0
Crib licking					
*Aronia melanocarpa* (LF)	60	0.5 ^Aabc^	0.62	0.0	2.0
*Carum carvi* (LF)	60	0.3	0.52	0.0	2.0
*Rubus idaeus* (LF)	60	0.1 ^A^	0.22	0.0	1.0
*Rosa canina* (SM)	60	0.3	0.57	0.0	2.0
*Calendula officinalis* (SM)	60	0.1 ^a^	0.32	0.0	1.0
*Salvia rosmarinus* (SM)	60	0.3	0.54	0.0	2.0
*Achillea millefolium* (ST)	60	0.3	0.49	0.0	2.0
*Origanum vulgare* (ST)	60	0.1 ^b^	0.47	0.0	3.0
*Leonurus cardiaca* (ST)	60	0.2 ^c^	0.38	0.0	1.0
Mouth licking					
*Aronia melanocarpa* (LF)	60	0.1	0.39	0.0	2.0
*Carum carvi* (LF)	60	0.1	0.34	0.0	1.0
*Rubus idaeus* (LF)	60	0.0	0.00	0.0	0.0
*Rosa canina* (SM)	60	0.1	0.32	0.0	1.0
*Calendula officinalis* (SM)	60	0.1	0.31	0.0	2.0
*Salvia rosmarinus* (SM)	60	0.1	0.25	0.0	1.0
*Achillea millefolium* (ST)	60	0.1	0.35	0.0	2.0
*Origanum vulgare* (ST)	60	0.1	0.30	0.0	1.0
*Leonurus cardiaca* (ST)	60	0.1	0.22	0.0	1.0
Empty-mouth chewing					
*Aronia melanocarpa* (LF)	60	0.3	0.59	0.0	2.0
*Carum carvi* (LF)	60	0.2	0.49	0.0	2.0
*Rubus idaeus* (LF)	60	0.3	0.48	0.0	2.0
*Rosa canina* (SM)	60	0.3	0.52	0.0	2.0
*Calendula officinalis* (SM)	60	0.2	0.46	0.0	2.0
*Salvia rosmarinus* (SM)	60	0.1	0.32	0.0	1.0
*Achillea millefolium* (ST)	60	0.3	0.55	0.0	2.0
*Origanum vulgare* (ST)	60	0.3	0.53	0.0	2.0
*Leonurus cardiaca* (ST)	60	0.2	0.45	0.0	2.0

n—number of observations (repeated measures); SD—standard deviation; LF—least familiar plants; SM—plants known by smell; ST—plants known by smell and taste. Differences between means in columns are statistically significant: they are marked with the same small letters (^a^, ^b^, ^c^) at *p* < 0.05 and with the same capital letters (^A^) at *p* < 0.01. Letters refer to GLMM LSMeans comparisons.

## Data Availability

The data presented in this study are available on request from the corresponding author.

## Abbreviations

The following abbreviations are used in this manuscript:

LF	Plants least familiar to horses
SM	Plants known by smell
ST	Plants known by smell and taste
